# Parkinsonism associated with multiple sclerosis: A report of eight new cases and a review on the literature

**Published:** 2014-04-03

**Authors:** Masoud Etemadifar, Farshid Afshar, Zahra Nasr, Mohsen Kheradmand

**Affiliations:** 1 Department of Neurology, School of Medicine, Isfahan Neurosciences Research Center, Isfahan University of Medical Sciences, Isfahan, Iran; 2 Department of Medical Sciences, School of Medicine, Medical Students’ Research Center, Isfahan University of Medical Sciences, Isfahan, Iran

**Keywords:** Extrapyramidal Signs, Magnetic Resonance Imaging, Movement Disorders, Multiple Sclerosis, Parkinsonism

## Abstract

**Background:** Multiple sclerosis (MS) is an autoimmune inflammatory disease that affects the central nervous system. Except tremor, other movement disorders such as Parkinsonism are less frequent in MS. However, some investigations have shown inflammatory and autoimmune aspects of Parkinson’s disease. In this study, we report eight new cases of MS who present Parkinson’s disease.

**Methods:** This retrospective population-based study was carried out on Isfahan MS society between April 2003 and July 2012. A total of 3792 patients with MS were surveyed for Parkinson’s disease. Parkinson’s disease was approved according to “UK Parkinson disease Brain Bank” criteria. Eligible cases were invited to have an interview with a neurologist. MRI was carried out according to the baseline.

**Results:** We identified eight patients (three men and five women) who present MS and Parkinson’s disease. The mean (±SD) age of onset was 33.3 ± 6.5 (range: 24–42) years for MS and 39.5 ± 8.6 (range: 30–55) years for Parkinson’s disease patients. In all patients, MS was preceded Parkinson’s disease. Parkinson’s disease was developed within mean duration of 6.1 ± 3.4 (range 2–13) years after MS. Focal lesions was shown in six cases, lesions of basal ganglia (BG) in four, lesions of the thalamus in two and lesions of mid-brain in five of cases. In four cases, Parkinsonism occurred before age 40 that is considered as Young-Onset Parkinson’s disease.

**Conclusion:** We reported eight patients with MS and Parkinson’s disease. To the best of our knowledge, 34 cases of Parkinsonism associating with MS are reported so far. Parkinsonism is a movement disorder, defines as deep gray matter disorder which lead to dopamine deficiency in BG. Investigations have shown that MS could affect deep gray matter structures. Demyelinated lesions in MS and consequence axonal loss in BG and/or nigrostriatal pathway may be responsible for Parkinsonism manifestations in such cases.

## Introduction

Multiple sclerosis (MS) is an autoimmune inflammatory disease, which commonly affects the central nervous system (CNS). MS has a wide range of manifestations, depending on the location of lesions. Although tremor is one of the frequent symptoms, other movement disorders are uncommon in patients with MS (accounts only in 1.6% of patients).^1^ Parkinsonism is a movement disorder with variable etiologies, defined as deep gray matter disarrange, caused by destruction of neurons in the substantia nigra (SN), which lead to dopamine deficiency in basal ganglia (BG). One of the most frequent reasons of Parkinsonism is Parkinson disease (PD), which occurs in almost 80% of cases with Parkinsonim.^2^ Some studies have suggested that inflammation and crossing of immune cells to the CNS may play a role in Parkinson’s disease.^3^ On the other hand, it was thought that MS is a disease of white matter. Now-a-days, due to advances in medical imaging, presence of lesions in cortical and subcortical gray matter has been proved.^4^ Number of reported cases with both MS and Parkinsonism is limited until now. It still remains unclear whether there is a “cause and effect” relationship between MS and Parkinsonism or they are just different disorders with different pathogenesis that randomly occur in an individual. In this research, we report eight new PD cases and review previous reports of both MS and Parkinsonism associated patients.

## Materials and Methods

This retrospective based-population study has been performed in Isfahan MS Society (IMSS) between April 2003 and July 2012. Isfahan is one of the metropolises of Iran. The recorded data of 3972 patients with definite MS - according to 2005 McDonald revised criteria^5^ - was available in the medical records of this society. Demographic and clinical features of these patients are available in another previous report.^6^

IMSS is the most major center includes approximately all of the MS patients of the province. To benefit from the remedial, consulting, and supporting services, MS patients must register in this society. Furthermore, it is requested from all the neurologists in Isfahan to refer their new detected MS cases to IMSS. However, we cannot ignore the possibility of few patients who prefer to private care and do not register in this society. Complete demographic data, family history, clinical, and medication history of MS patients are recorded in IMSS. According to the standardized MRI protocol for MS, MRI is carried out for patients.^7^

MRI of patient’s in this society has been performed in these special times: initially when a patient is suspicious for MS, if the disease becomes worse in an unexpected manner, and if there are some manifestations, which lead us to another diagnosis. The MRI reports include the number and location of T2-hyperintense and T1-hypointense lesions and atrophies. We searched for patients with manifestations of PD from definite MS cases in IMSS and found eligible cases. Then, we invited these patients to have an interview with a neurologist. Subjects were provided with explanations about the details of the study protocol and written informed consent was obtained. Their PD was readmitted by an experienced neurologist (ME) and needed clinical information of these patients has been revised. Diagnosis of PD was established on the existence of bradykinesia, rigidity, resting tremor, postural instability and increasing in severity of disorder and also given the unilateral or asymmetrical nature of symptoms, for 10 years or more (according to “UK PD Brain Bank” criteria).^8^ We also used this criteria to differentiate and exclude PD patients from Parkinson plus and secondary Parkinsonism ones. This study was approved by our local Ethics Committee. Furthermore, the occurrence of PD before the age of 40 is considered as Young-onset Parkinson disease (YOPD).^9^

## Results

In this study, we identified eight MS patients (five women and three men) presented with the symptoms of PD. Demographic and clinical details of patients are summarized in table 1. The mean (±SD) age of onset was 33.3 ± 6.5 (females: 32.6 ±± 7, males: 34.6 ± 6.8) with the range of 24–42 years for MS and 39.5 ± 8.6 (females: 39.8 ± 9.9, males: 39 ± 7.8) with the range: 30–55 years for PD patients. In all cases, MS was preceded PD. The mean of expanded disability status scale (EDSS), which obtained after administrations of drugs was 4.6 ± 0.8. PD was developed within mean duration of 6.1 ± 3.4 (range 2–13) years after MS. None of the patients had a family history of MS and PD. The clinical MS course of all cases was secondary progressive (SP). The mean of MS relapse rate (RR) before developing PD was 3.6 ± 1.3, where these relapses occurred in average 6.1 ± 3.4 years (RR/years: 0.7). The mean of MS relapse rate after developing PD was 0.7 ± 0.7 which occurred in average 4.8 ± 3.7 years (RR/years: 0.3). In four cases, (1, 4–6) PD occurred as YOPD. According to MRI findings, strategically located lesions was observed in six cases (1, 2, 4–6, 8), lesions of BG in four cases (2, 4–6), lesions of the thalamus in two cases (1, 2), and lesions of midbrain in five cases.

## Discussion

PD is an age-related disorder; defined by deep gray matter abnormality. Malfunction in the motor loop of the BG loop leads to Parkinsonism. Motor circuit includes SN, neostriatum, subthalamic nucleus, globus pallidus, and motor nuclei of thalamus (mostly ventral anterior and ventrolateral nuclei).^10^ Lesions of the BG could cause Parkinsonism manifestations.^11^ Studies have shown that impairment of the blood–brain barrier, infiltration of the peripheral immune within this barrier and finally, inflammation in the CNS have important roles in PD pathogenesis,^3^ similar to what happens in MS. One of the supporting evidences implying on this claim is the reduction of PD developing risk in individuals who are taking anti-inflammation drugs.^12^ Notably, some studies suggest peripheral immune cells are responsible for progressive feature of this disorder.^13^

**Table 1:** Demographic and clinical findings of patients with MS and PD

**Case**	**1**	**2**	**3**	**4**	**5**	**6**	**7**	**8**
**Sex**	**Male**	**Female**	**Male**	**Female**	**Female**	**Female**	**Male**	**Female**
Age of the patient	32	60	49	41	41	42	45	45
Age of onset (MS)	27	42	40	31	29	24	37	37
Age of onset (PD)	30	55	44	39	31	31	43	43
Initial presentation (MS)	Ataxia	Ataxia, leg weakness	Leg weakness	Facial palsy	Ataxia	Visual loss	Diplopic, vertigo	Ataxia
MS manifestations (during course of the disease)	Spasticity, epilepsy	Spasticity	Spasticity	Nystagmus, epilepsy	Epilepsy	Epilepsy	Spasticity, nystagmus	Spasticity, seizure
RR before PD	2	3	4	3	3	5	3	6
RR after PD	0	1	1	1	0	0	1	2
PD symptoms	Resting tremor, rigidity, bradykinesia	Rigidity, bradykinesia, amnesia	Cervical dystonia, rigidity, bradykinesia	Rigidity, bradykinesia, dementia	Cervical dystonia, rigidity, bradykinesia, dementia	Cervical dystonia, rigidity, bradykinesia, dementia	Resting tremor, rigidity, bradykinesia	Resting tremor, rigidity, bradykinesia, dementia
MRI findings	PV, MD, TH	PV, C, JC, TH, BG, atrophy	PV, atrophy	PV, C, JC, MD, BG	PV, BG, JC, C, MD	PV, BG, CC, C, MD	PV, JC	PV, JC, MD
Drugs	Levodopa, imuran, amantadine	Levodopa, methotrexate, amantadine	Levodopa, methotrexate, amantadine	Levodopa, methotrexate, amantadine	Levodopa, imuran, amantadine, betaferon	Levodopa, amantadine, selegiline	Levodopa, methotrexate, amantadine	Levodopa, amantadine
Last EDSS	4.5	5	5	5.5	4	6	3.5	4

PV: Periventricular; C: Cortex; CC: Cerebellar cortex; MD: Midbrain; JC: Juxtacortical; TH: Thalamus; BG: Basal ganglia; BS: Brain stem; RR: Relapse rate; EDSS: Expanded disability status scale; MS: Multiple sclerosis; PD: Parkinson disease; MRI: Magnetic resonance imaging

MS formerly was supposed as a white matter demyelinating disease because of difficulty of gray matter imaging. Now-a-days, it has been proved that due to the existence of myelinated fibers in gray matter, MS can affect cortical/subcortical gray matter; especially in chronic phases of the disease. Furthermore, low contrast between demyelination areas and normal tissues because of low density of myelin in gray matter lead to the existence of invisible lesions, which cannot relieve with usual MRI in most cases. More sensitive MRI methods (double inversion recovery) show gray matter lesions five times more than common conventional MRI method. However, almost 80% of gray matter lesions detected by microscopic examinations cannot be observed either by this technique. Studies demonstrated that gray matter lesions are common in all clinical courses of MS. Furthermore in some cases, gray matter lesions are formed earlier than white matter ones.^4^

In our study, six cases have symptomatic lesions and seven (1, 3-8) showed Parkinsonism signs before the age of 50. PD is diagnosed rarely in individuals under 50-year old.^9^ In four cases, (4–6, 8) Dementia was seen at the process of PD. Although dementia is rare in MS, it is a common feature of PD.^14^^,^^15^ The clinical MS course of all cases was SP while the prevalence of SP type is only 6.4% among MS patients in Isfahan.^6^ We assume at least in six of them probably there is a causal relationship due to the existence of symptomatic lesions. However, this possibility should also be considered that there had been some previous resolved lesion in a strategic location that could not be detected by MRI at the time of study. The mean EDSS obtained after drug administration was 4.6 ± 0.8. When compared with the mean of MS patients EDSS (2.7 ± 1.8) in Isfahan, it is higher.^6^ Great number of EDSS among our cases and in previous reports could be related to progressive type of MS (primary progressive [PP]/SP) or maybe due to postural instability and ambulatory difficulty, which is one of Parkinsonism symptoms. Of great importance, disability in MS is relatively correlated with gray matter in compare with white matter.^4^ Therefore, the more destruction in gray matter results a significant rise in EDSS number. SPMS begins as RRMS; however, after a while it turns into SP. Thus, in the early stage of disease, amount of attacks are greater. Unfortunately, we did not have the follow-up of the patients associated with their responses to corticosteroids and levodopa. In addition, lack of genetic experiments and not using complimentary neuroimaging methods [dopamine transporters (DaT) scan and positron emission tomography scan] are other limitations of our study.

To the best of our knowledge, 34 cases with simultaneity of MS and Parkinsonism have been reported until now (Table 2).^16^^-^^35^ Details of each case are shown in table 2. It is not clear whether there is a causal relationship between MS and PD or not. There is a probability that MS plaques can affect BG or other structures having an important role in nigrostriatal pathway and lead to dysfunction of extrapyramidal pathway and cause Parkinsonism.^36^^,^^37^

Sadnicka et al. focused on genetic aspects of the relation between MS and PD by reporting a patient having these two disorders with a heterozygous mutation in parkin gene.^27^ Mutation of the gene called “parkin” is associated with PD. Furthermore, expression of this gene increases in acute plaques of MS.^38^ PINK1 also has the same features. 

Wilhelmus et al. showed marked astrocytic PINK1 immunostaining in demyelination lesions of MS and suggested that PINK1 is associating in limiting of cellular injury as a protective factor.^39^ Another supporting evidence for this relationship is the genetic variability of HLA-DRB5 which has a role in inflammatory aspects of both MS and PD.^40^

Despite the existence of symptomatic demyelination lesions in some cases (15, 17), in order to previous reports, the focal lesions can cause levodopa-responsive and levodopa-induced only in little cases.^41^ Therefore, it is possible that these lesions are not associated with the emergence of Parkinsonism (2). Although Parkinsonism occurs rarely among MS patients, appearing BG and thalamus lesions is a common phenomenon in the MRI of MS patients.^36^ This is in keeping with reports that implies on weak relationship between locations of lesions and clinical manifestations.^42^ Due to this, probably lesions in BG may not play a role in appearance of Parkinsonism. Based on the above description, 13 cases seems to have two coincidental disease rather than two related disease (3, 4, 8, 9, 15-17, 19, 21–23, 25, 27). In some cases, (16, 17) Parkinsonism occurred when MRI of patients were normal and they do not have any signs of MS abnormalities. After a while, since clinical manifestations of PD have been developed, MS manifestations appeared. Although, after initiation of MS, symptomatic lesions appeared, these lesions cannot be considered as a cause of Parkinsonism. In the most likely state, MS lesions may be responsible for rapid aggravation of PD.^20^ In case 15, first manifestation of PD starts a long time after the onset of MS, at the age of 51. Forasmuch as PD is an age-related disease which its incidence increases markedly with aging, maybe PD occurred as its routine pathogenesis. The window time between initiation of neurons degeneration and presenting the first manifestation of PD is not clear yet. Some realizations suggest that long time (several decades) is needed; while some others say 5 years or less.

## Conclusion

Although concomitancy of MS and PD were suggested because of casual relationship or as an accident, limited studies on this issue do not support the accidental occurrence of MS and PD. Cases which were reported until now represent both casual and accidental aspect. Moreover, the exact etiology of MS and PD still remains unclear and put more weight for difficulty of explanation and results in controversial hypothesizes. Further pathophysiological studies are needed to clarify different aspects.

**Table 2:** Cases with simultaneity of MS and Parkinsonism

**Author, year**	**Case**	**Sex**	**Family history (PD and MS)**	**MS type**	**MRI lesions** *	**DaT scan findings**	**Last EDSS**	**Corticosteroid response**	**Levodopa response**
Fog and Linnemann.^34^	1	Female	UN	UN	UN	UN	UN	UN	UN
	2	Female	UN	UN	UN	UN	UN	UN	UN
Mao et al.^19^	3	Female	UN	UN	-	UN	UN	-	+
	4	Female	-	UN	-	UN	UN	-	+
Vieregge et al.^33^	5	Male	-	UN	Near lat TH and GP	UN	UN	+	UN
	6	Female	-	UN	Near lat TH and LF	UN	UN	+	-
Tranchant et al.^31^	7	Female	UN	UN	CP near SN	UN	UN	UN	UN; two of these three cases had positive response of levodopa
	8	Male	UN	UN	-	UN	UN	UN	
	9	Male	UN	UN	-	UN	UN	UN	
Maranhao et al.^18^	10	Male	UN	UN	CP, TH, GP	UN	UN	UN	UN
Burn and Cartlidge.^25^	11	Female	UN	UN	-	UN	UN	+	-
Federlein.^22^	12	Male	UN	UN	Near SN	UN	UN	+	UN
Wittstock et al.^35^	13	Female	UN	UN	SN	UN	UN	+	+
Folgar et al.^21^	14	Female	-	RR	-a	UN	UN	+	UN
Ozturk et al.^16^	15	Female	-	RR	MS including SN	UN	UN	UN	+
Kreisler et al.^20^	16	Female	+ (PD and MS)	UN	SN	UN	UN	-	+
Valkovic et al.^30^	17	Male	-	UN	Sub thalamic region	Abnormal	UN	UN	+
Barun et al.^26^	18	Female	UN	RR	-	Abnormal	UN	+	+ (simultaneously with corticosteroid)
	19	Female	UN	RR	-	Abnormal	UN	UN	+
Nociti et al.^26^	20	Female	UN	SP	TH, BG	UN	UN	-	UN
	21	Male	UN	RR	-	UN	UN	-	UN
	22	Female	UN	RR	-	UN	UN	-	+
Delgado et al.^23^	23	Female	UN	PP	-	UN	8.5	-	-
Saidha et al.^29^	24	Male	UN	UN	SN, GP, TH	UN	UN	+	-
Damasio et al.^24^	25	Female	-	RR	-	Abnormal	UN	-	+
Schultheiss et al.^28^	26	Male	UN	PP	BG	UN	6.5	-	-
Sadnicka et al.^27^	27	Male	-	PP	-	Abnormal	UN	-	+
Pedemonte et al.^32^	28	Female	UN	RR	UN	Abnormal	3	UN	+
	29	Female	UN	RR	UN	Abnormal	6	UN	+
	30	Female	UN	SP	UN	Abnormal	6.5	UN	+
	31	Female	UN	SP	UN	UN	7	UN	+
	32	Female	UN	PP	UN	UN	5.5	UN	+
	33	Male	UN	RR	UN	UN	5	UN	+
	34	Female	UN	PP	UN	Abnormal	6.6	UN	+

GP: Globus pallidus; SP: Secondary progressive; PP: Primary progressive; RR: Relapse rate; EDSS: Expanded disability status scale; MRI: Magnetic resonance imaging; PD: Parkinson disease; SN: Substantia nigra; MS: Multiple sclerosis; CP: Cerebral peduncles; TH: Thalamus; BG: Basal ganglia; UN: Unknown, not tried, not used, not mentioned or unclear details.

* Only lesions that may have been related to Parkinsonism signs; not all lesions.

a Last scan was performed before Parkinsonism manifestations
